# Real-world outcomes of newly diagnosed AML treated with venetoclax and azacitidine or low-dose cytarabine in the UK NHS

**DOI:** 10.1016/j.bneo.2024.100017

**Published:** 2024-05-23

**Authors:** Jad Othman, Ho Pui Jeff Lam, Sarah Leong, Faisal Basheer, Islam Abdallah, Kathryn Fleming, Priyanka Mehta, Heba Yassin, John Laurie, Michael Austin, Paolo Gallipoli, Tom Taylor, Mike Dennis, Johnathon Elliot, Georgina Clarke, Raymond Dang, Jennifer Vidler, Pramila Krishnamurthy, Anne-Louise Latif, Pallavi Kalkur, Maryam Shahidianakbar, Victoria Campbell, Deepak Mannari, Emily Sutherland, Thishakya Wickramaratne, Angela Collins, Rui Zhao, Herng Mak, Edward Belsham, Shabnam Banerjee, Jamila Bashir, Srinivas Pillai, Richard Whitmill, Sofia Galli, Mariam Amer, Vidhya Murthy, Duncan Murray, Farooq Wandroo, Francesca Hogan, Francesca Crolla, Nicole Fowler, Anjum Khan, Jenny O’Nions, Richard Dillon

**Affiliations:** 1 Department of Medical and Molecular Genetics, King’s College London, London, United Kingdom; 2 Department of Haematology, Guy’s and St Thomas’ NHS Foundation Trust, London, United Kingdom; 3 Faculty of Medicine and Health, University of Sydney, Sydney, Australia; 4 University College London Hospital NHS Foundation Trust, London, United Kingdom; 5 Department of Haematology, Addenbrooke’s Hospital, Cambridge, United Kingdom; 6 Department of Haematology, Leeds Teaching Hospitals Trust, Leeds, United Kingdom; 7 University Hospital Bristol, Bristol, United Kingdom; 8 University Hospitals Sussex NHS Foundation Trust, Worthing, United Kingdom; 9 Barts Cancer Institute, Queen Mary University of London, London, United Kingdom; 10 Nottingham University Hospital, Nottingham, United Kingdom; 11 The Christie NHS Foundation Trust, Manchester, United Kingdom; 12 James Cook University Hospital, Middlesbrough, United Kingdom; 13 King’s College Hospital, London, United Kingdom; 14 Department of Haematology, Queen Elizabeth University Hospital, Glasgow, United Kingdom; 15 Southend University Hospital, Southend-on-sea, United Kingdom; 16 Mersey and West Lancashire Teaching Hospitals NHS trust, Whiston, United Kingdom; 17 Western General Hospital, Edinburgh, United Kingdom; 18 Musgrove Park Hospital, Somerset NHS Foundation Trust, Taunton, United Kingdom; 19 City Hospitals Sunderland NHS Trust, Sunderland, United Kingdom; 20 Blackpool Teaching Hospitals NHS Foundation Trust, Blackpool, United Kingdom; 21 Norfolk and Norwich University Hospitals NHS Foundation Trust, Norwich, United Kingdom; 22 Torbay Hospital, Torquay, United Kingdom; 23 University Hospitals Sussex NHS Foundation Trust, Brighton, United Kingdom; 24 Portsmouth Hospitals University NHS Trust, Portsmouth, United Kingdom; 25 Queens Hospital, Barking, Havering and Redbridge University Hospitals NHS Trust, Romford, United Kingdom; 26 University Hospitals of Derby and Burton NHS Foundation Trust, United Kingdom; 27 Royal Stoke University Hospital, University Hospital of North Midlands NHS Trust, Stoke-on-Trent, United Kingdom; 28 New Cross Hospital, The Royal Wolverhampton NHS Trust, Wolverhampton, United Kingdom; 29 Frimley Park Hospital, London, United Kingdom; 30 University Hospital Southampton, Southampton, United Kingdom; 31 Centre for Clinical Haematology, University Hospitals Birmingham, Birmingham, United Kingdom; 32 University Hospitals Coventry and Warwickshire NHS Trust, Coventry, United Kingdom; 33 Sandwell and West Birmingham Hospitals NHS Trust, Birmingham, United Kingdom; 34 University Hospital of Wales, Cardiff, United Kingdom; 35 University Hospitals Plymouth NHS Trust, Plymouth, United Kingdom; 36 Royal Cornwall Hospitals NHS Trust, Truro, United Kingdom

## Abstract

Venetoclax with azacitidine is the standard of care for patients with acute myeloid leukemia (AML) who are unfit for intensive chemotherapy; however, uncertainties remain regarding the treatment schedule, accurate prognostication, and outcomes for patients treated outside clinical trials. The option of venetoclax with low-dose cytarabine (LDAC) is also available; however, it is not clear for which patients it may be a useful alternative. Here, we report a large real-world cohort of 654 patients treated in 53 UK hospitals with either venetoclax and azacitidine (n = 587) or LDAC (n = 67). The median age was 73 years, and 59% had de novo AML. Most patients received 100 mg of venetoclax with an azole antifungal. In cycle 1, patients spent a median of 14 days in the hospital, and 85% required red cell transfusion, 59% platelet transfusion, and 63% required IV antibiotics. Supportive care requirements significantly reduced after the first cycle. Patients receiving venetoclax-azacitidine had a complete remission (CR)/CR with incomplete hematological recovery rate of 67%, day 30 and day 60 mortality of 5% and 8%, respectively, and median overall survival of 13.6 months. Mutations in *NPM1*, *RUNX1*, *STAG2*, and *IDH2* were associated with improved survival, whereas age, secondary and therapy-related AML, +8, *MECOM* rearrangements, complex karyotype, *ASXL1*, and *KIT* mutations were associated with poorer survival. Prognostic systems derived specifically for patients treated with venetoclax-azacitidine performed better than the European LeukemiaNet and Medical Research Council classifications; however, improved risk classifications are still required. In the 149 patients with *NPM1* mutated AML, outcomes were similar for those treated with venetoclax-azacitidine and venetoclax-LDAC.

## Key Points

• Outcomes of patients treated with venetoclax-based nonintensive therapies across >50 NHS hospitals mirror those seen in clinical trials.
• Current mutation based prognostic systems are inadequate; collaborative efforts are needed to establish a definitive prognostic scheme.

## Visual Abstract

**Figure:**
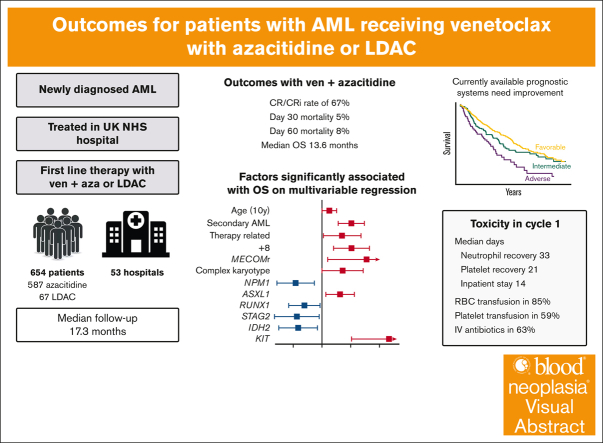

## Introduction

The incidence of acute myeloid leukemia (AML) increases with age, and almost half of diagnoses are made in patients aged >70 years.^1^ Outcomes remain suboptimal in these older patients, most of whom are not candidates for intensive chemotherapy. The BCL2 inhibitor venetoclax was the first new therapy in almost 2 decades to demonstrate an improvement in survival for these patients. In the VIALE-A study, venetoclax combined with azacitidine improved the median overall survival (OS) from 9.6 to 14.7 months, compared with placebo.^2^ When combined with low-dose cytarabine (LDAC) in the VIALE-C study, venetoclax improved the median OS from 4.1 to 8.4 months.^3^ Since the publication of these studies, venetoclax with azacitidine has become the standard of care for patients with AML unfit for intensive chemotherapy in many countries.

Because of the relatively strict inclusion criteria for these trials, which mainly recruited patients from large academic centers, there remains uncertainty about the outcomes of patients treated with venetoclax-based regimens in routine clinical care. Additionally, unanswered questions remain regarding the optimal use of these therapies. The recommended dose is 400 mg daily for 28 days in each cycle; however, clinicians are increasingly using shorter regimens and lower doses in combination with azole antifungals.^4^, ^5^, ^6^ Whether outcomes will be similar with these modifications is not established. Accurate prognostication also remains difficult in patients treated with venetoclax regimens. Established AML risk classifications are derived from intensively treated cohorts and do not provide accurate prognostic information in patients treated with lower-intensity therapy.^7^, ^8^, ^9^ Although a number of alternative prognostic systems have been proposed, these have not been sufficiently validated in large patient cohorts. Finally, although better outcomes were reported in VIALE-A than VIALE-C, LDAC is used as the backbone in some jurisdictions, but no real-world outcomes have been reported, and it remains unclear whether venetoclax-cytarabine could be an appropriate treatment for some patients.

A number of groups have reported real-world outcomes with venetoclax and azacitidine; however, these have mostly been single-center series from large academic medical centers and have included relatively small numbers of patients.^8^^,^^10^, ^11^, ^12^, ^13^, ^14^, ^15^, ^16^, ^17^, ^18^, ^19^ In the United Kingdom, venetoclax was first made available in August 2020 in response to the coronavirus pandemic and subsequently approved for routine use in patients unsuitable for intensive chemotherapy. Here, we present a health system–wide analysis of a large cohort of patients with AML treated with venetoclax and either azacitidine or LDAC in the UK National Health Service (NHS) including patients treated at 53 hospitals ranging from large academic centers to smaller district general hospitals. We describe the treatment administered, supportive care requirements, remission rates, survival, and clinical and genomic factors associated with outcomes in this real-world setting.

## Methods

### Patients

Patients were included in this study if they had newly diagnosed AML and received venetoclax with either azacitidine or LDAC. They could not have received prior therapy for AML apart from hydroxycarbamide or similar for cytoreduction, but patients who had previously received therapy for myelodysplastic syndrome (MDS) or other hematological conditions were included.

Sites were invited to participate by an email from the study team, which was sent to all hospitals who requested permission to prescribe venetoclax from a central NHS system. Participating centers were asked to include all patients treated at their site during the data collection period. Data were collected retrospectively by clinicians or research staff, anonymized, and entered into a central REDCap database. Venetoclax dose, duration, and toxicity information was requested for the first 4 cycles of therapy. After an initial phase performed as a service evaluation in July 2021, subsequent data were collected as part of a project approved by the Central Bristol Research Ethics Committee (22/SW/0042). Outcomes of a subset of patients in this analysis have been previously reported.^20^

### Treatment

Venetoclax was made available as an emergency measure during the COVID-19 pandemic in April 2020, aiming to reduce both mortality and health care resource use associated with intensive chemotherapy. Guidance provided to clinicians at the time is shown in the supplemental Methods. Venetoclax with azacitidine and venetoclax with LDAC were approved by The National Institute for Health and Care Excellence in February and April 2022, respectively, for use in the United Kingdom for patients unsuitable for intensive chemotherapy, as per the marketing authorization. Patients treated via both access schemes were included in the study.

Treatment was as per institutional guidelines. The initial guidance provided for the COVID-19 emergency access was for venetoclax at 100 mg on day 1, 200 mg on day 2, and 300 mg on day 3, followed by 100 mg a day combined with posaconazole or voriconazole from days 4 to 28. A bone marrow biopsy was suggested between days 21 and 28. The number of days of venetoclax in subsequent cycles could be reduced based on response, cytopenias, and local guidelines. The choice between azacitidine and LDAC was made by the treating clinician, with LDAC recommended as an acceptable alternative for patients with *NPM1* or *IDH1/2* mutated disease.

### End points and statistical methods

Remissions were defined as per European LeukemiaNet (ELN) 2017 criteria,^21^ and assigned by the treating clinician. OS was calculated from day 1 of cycle 1 until the day of death, censored on the date last known to be alive. Cumulative incidence of relapse was calculated for patients achieving complete remission (CR) or CR with incomplete hematological recovery (CRi), from the date of remission to relapse or death, with nonrelapse mortality as a competing risk. Neutrophil and platelet recovery was calculated as the number of days until neutrophils were consistently >0.5 × 10^9^/L and platelets >50 × 10^9^/L, from day 1 of therapy. If no counts below this level were observed, the day of recovery was recorded as day 0.

Cytogenetics, *FLT3, NPM1*, and next-generation sequencing (NGS) gene panel testing were performed at local or regional laboratories, as deemed appropriate by the treating clinician. If a karyotype was unevaluable, this was considered to be intermediate risk for Medical Research Council (MRC) and ELN 2022 risk assignment. Complex karyotype was assigned using ELN 2022 criteria (≥3 changes in the absence of recurring genetic abnormalities, not including hyperdiploid karyotypes). For classification of AML into the World Health Organization (WHO) and International Consensus Classification (ICC) categories, *CEBPA* was not considered because information was not uniformly available on the number and location of mutations, and any *TP53* mutation was included because information on variant allele fraction (VAF) was also not routinely entered.

Factors associated with achievement of remission and OS were analyzed using binomial logistic and Cox regression, respectively. Age was included in 10-year intervals, and platelet and white cell count (WCC) were log transformed. Multivariable regression was performed including baseline factors present in >3% of patients and factors significant on univariable analysis. Due to the amount of missing data for baseline blood counts (290 patients) and performance status (142 patients), these were not included in multivariable assessment. Complex karyotype was colinear with −5/del5q, −7/abn7q, and −17/abn17p. To address this in the multivariable regression, patients with complex karyotype were assigned to this category only, whereas −5/del5q, −7/abn7q, and −17/abn17p were only assigned when occurring in the absence of complex karyotype. Normal karyotype was not included in the multivariable analysis due to being mutually exclusive of all other cytogenetic abnormalities. Missing data were not imputed. All analyses were performed with R statistical software version 4.3.2.

## Results

### Patient characteristics

A total of 654 patients were included from 53 hospitals, 587 treated with venetoclax and azacitidine and 67 with venetoclax and LDAC. A median of 9 patients were included per hospital (range, 1-104; supplemental Figure 1), with a median of 0.59 patients (interquartile range [IQR], 0.42-0.88) starting treatment per month per hospital. The median follow-up by reverse Kaplan-Meier method was 17.3 months.

The median age was 73 years (range, 16-90), and 60% were male (Table 1). A total of 387 patients (59%) had de novo AML, 202 (31%) were secondary to an antecedent hematological disorder, and 65 (9.9%) were therapy related. NGS results were available in 516 patients. The most frequently mutated genes were *NPM1* (24%), *SRSF2* (24%), *ASXL1* (23%), *TET2* (22%), *RUNX1* (21%), *DNMT3A* (18%), *IDH2* (17%), *TP53* (13%), and *FLT3* internal tandem duplication (ITD, 11%). The most common WHO/ICC disease classifications were AML, MDS-related (49%), and AML with *NPM1* mutation (25%).

**Table 1.** **Baseline characteristics**

Characteristic	All patients, n = 654	Azacitidine, n = 587	LDAC, n = 67
Median age (IQR)	73 (68-76)	73 (68-76)	73 (68-77)
Female	261 (40%)	230 (39%)	31 (46%)
**Clinical disease type**			
De novo	387 (59%)	341 (58%)	46 (69%)
Secondary	202 (31%)	186 (32%)	16 (24%)
MDS	107 (53%)	98 (53%)	9 (56%)
MDS/MPN	40 (20%)	35 (19%)	5 (31%)
MPN	44 (22%)	42 (23%)	2 (13%)
Other or unknown	11 (5.5%)	11 (5.9%)	0 (0%)
Therapy related	65 (9.9%)	60 (10%)	5 (7.5%)
**Baseline blood counts**			
WCC, median (IQR)	5 (2-22)	5 (2-19)	14 (2-62)
Hemoglobin, median (IQR)	87 (75-101)	87 (75-101)	88 (78-100)
Platelet count, median (IQR)	71 (35-123)	74 (36-124)	55 (32-93)
Missing	289	264	25
Bone marrow blast, median (IQR), %	42 (25-70)	41 (25-70)	52 (30-79)
Missing	286	257	29
**Cytogenetic/FISH abnormalities**			
Core binding factor fusions	5 (0.8%)	5 (0.9%)	0 (0%)
+4	16 (2.6%)	16 (2.9%)	0 (0%)
+8	66 (11%)	61 (11%)	5 (7.8%)
+11	15 (2.5%)	14 (2.6%)	1 (1.6%)
+13	19 (3.1%)	18 (3.3%)	1 (1.6%)
+19	11 (1.8%)	11 (2.0%)	0 (0%)
+21	16 (2.6%)	15 (2.7%)	1 (1.6%)
del9q	4 (0.7%)	3 (0.5%)	1 (1.6%)
*KMT2A* rearrangement	8 (1.3%)	8 (1.5%)	0 (0%)
t(9;22)	2 (0.3%)	1 (0.2%)	1 (1.6%)
*MECOM* rearrangement	12 (2.0%)	12 (2.2%)	0 (0%)
−5/del5q	59 (10%)	57 (11%)	2 (3.2%)
−7/abn7q	83 (14%)	81 (15%)	2 (3.2%)
−17/abn17p	40 (6.8%)	40 (7.7%)	0 (0%)
Complex karyotype	91 (15%)	89 (16%)	2 (3.1%)
Hyperdiploid karyotype	14 (2.3%)	13 (2.4%)	1 (1.6%)
Normal karyotype	313 (51%)	267 (49%)	46 (72%)
Missing	43	40	3
*FLT3*-ITD	68 (11%)	58 (10%)	10 (15%)
Missing	17	16	1
*FLT3* TKD	41 (6.6%)	36 (6.5%)	5 (7.9%)
Missing	33	29	4
*NPM1*	149 (24%)	112 (20%)	37 (56%)
Missing	22	21	1
**Mutations on NGS panel**			
*CEBPA*	33 (6.4%)	28 (6.0%)	5 (10%)
*ASXL1*	119 (23%)	108 (23%)	11 (22%)
*BCOR*	34 (6.6%)	34 (7.3%)	0 (0%)
*EZH2*	20 (3.9%)	18 (3.9%)	2 (4.0%)
*RUNX1*	107 (21%)	102 (22%)	5 (10%)
*SF3B1*	16 (3.1%)	15 (3.2%)	1 (2.0%)
*SRSF2*	124 (24%)	113 (24%)	11 (22%)
*STAG2*	44 (8.5%)	40 (8.6%)	4 (8.0%)
*U2AF1*	35 (6.8%)	33 (7.1%)	2 (4.0%)
*ZRSR2*	13 (2.5%)	11 (2.4%)	2 (4.0%)
*TP53*	67 (13%)	66 (14%)	1 (2.0%)
*IDH1*	46 (8.9%)	45 (9.7%)	1 (2.0%)
*IDH2*	88 (17%)	74 (16%)	14 (28%)
*NRAS*	47 (9.1%)	45 (9.7%)	2 (4.0%)
*KRAS*	22 (4.3%)	19 (4.1%)	3 (6.0%)
*DNMT3A*	91 (18%)	78 (17%)	13 (26%)
*TET2*	112 (22%)	97 (21%)	15 (30%)
*KIT*	6 (1.2%)	5 (1.1%)	1 (2.0%)
*JAK2*	33 (6.4%)	32 (6.9%)	1 (2.0%)
Missing	138	121	17

FISH, fluorescence in situ hybridization; MPN, myeloproliferative neoplasm.

### Therapy administered and supportive care requirements

**Figure 1.**
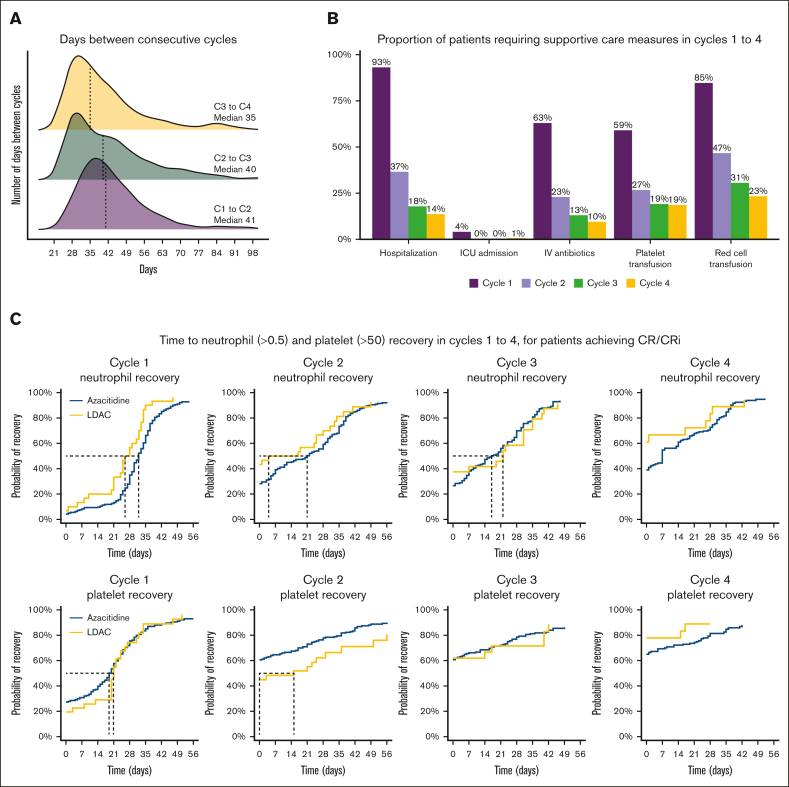
**Count recovery and supportive care requirements in cycles 1-4.** (A) Days between consecutive cycles. (B) Proportion of patients requiring supportive care measures in cycles 1 to 4. (C) Time to neutrophil and platelet recovery for patients who achieved CR/CRi.

Venetoclax dose and duration were available for 486 patients, of whom 405 also had data for count recovery and toxicity for cycles 1 to 4 (supplemental Table 1; supplemental Figure 2). The majority of patients (96%) received 100 mg of venetoclax with posaconazole or voriconazole. A median of 28 days venetoclax was prescribed to be administered in cycles 1 and 2 and 21 days in cycles 3 and 4. For patients receiving at least 4 cycles, the median days of venetoclax exposure during the first 4 cycles were 98 days (IQR, 84-112). The median duration between day 1 of a cycle and day 1 of the following cycle was 41 days (IQR, 35-50) for cycle 1, 40 days (IQR, 30-50) for cycle 2, and 35 days (IQR, 28-49) for cycle 3 (Figure 1A). Median hospital stay in cycle 1 was 14 days (IQR, 8-27), 85% of patients required red cell transfusion (median, 5 units), 59% required platelet transfusion (median, 1 unit), and 63% of patients required IV antibiotics. From cycle 2 onward, very few patients required hospitalization or transfusion (supplemental Table 1; Figure 1B; supplemental Figure 2).

Recovery of neutrophils to >0.5 × 10^9^/L, measured from day 1 of therapy, occurred at a median of 33 days (34 days with azacitidine and 26 days with LDAC; supplemental Table 3), with only 33% of patients recovering by day 28. Platelet recovery to >50 × 10^9^/L occurred at a median of 21 days (21 days azacitidine and 23 days LDAC), with 67% recovered by day 28. Hematological recovery in subsequent cycles occurred earlier, and platelet counts <50 × 10^9^/L were not recorded for most patients after cycle 2. When limiting the analysis only to patients achieving CR/CRi, the median time to neutrophil and platelet recovery in cycle 1 was 32 and 20 days, respectively (supplemental Table 2; Figure 1C).

Because the majority patients received venetoclax and azacitidine, and given the different patient characteristics of those receiving LDAC, we restricted the following analyses to patients receiving venetoclax with azacitidine.

### Achievement of remission with venetoclax and azacitidine

Response status was evaluable for 574 of the patients treated with venetoclax and azacitidine. A total of 272 (47%) achieved CR, and a further 113 (20%) achieved CRi, for a CR/CRi rate of 67% (Table 2). The best response was morphological leukemia-free state in 3.7% of patients and partial remission (PR) in 11%, with 11% having refractory disease and 7.7% dying before response assessment. As expected, survival outcomes were strongly determined by the best response achieved (supplemental Figure 3). Thirty-two patients (5.5%) received allogeneic transplant in first remission.

**Table 2.** **Remission and outcome**

Characteristic	Azacitidine, n = 587	LDAC, n = 67
**Best response**		
CR	272 (47%)	38 (58%)
CRi	114 (20%)	10 (15%)
Morphologic leukemia-free state	21 (3.7%)	0 (0%)
Partial remission	61 (11%)	1 (1.5%)
Refractory disease	62 (11%)	11 (17%)
Death before response assessment	44 (7.7%)	6 (9.1%)
Missing	13	1
Day 30 mortality	5%	6%
Day 60 mortality	8%	7%
**Allogeneic transplant**	35 (6.0%)	4 (6.0%)
In CR1	32 (5.5%)	3 (4.5%)
**OS**		
Median survival (mo)	13.6 (95% CI, 11.7-15.1)	10.9 (95% CI, 8.8-20.2)
12-mo survival	54%	46%

**Figure 2.**
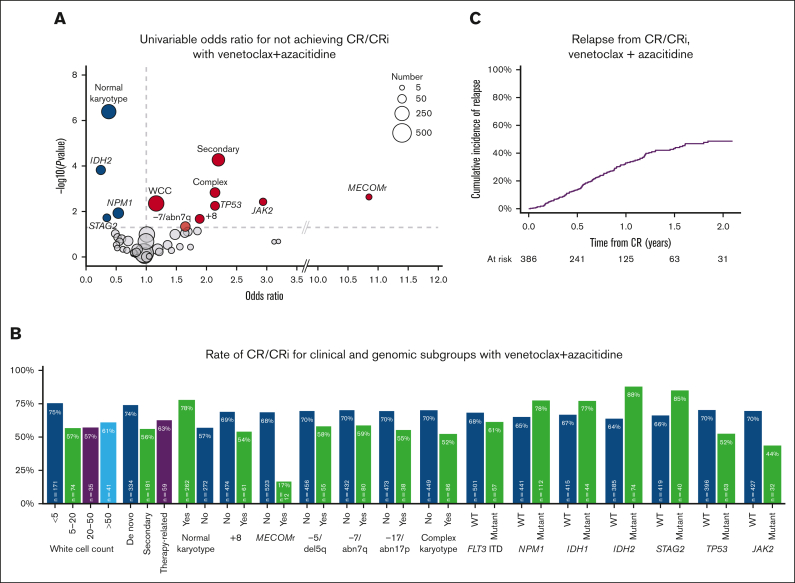
**Characteristics associated with achievement of remission with venetoclax and azacitidine.** (A) Univariable odds ratios for not achieving CR/CRi (factors with odds ratio <1 are associated with achievement of CR/CRi). (B) Rate of CR/CRi in clinical and genomic subgroups. (C) Cumulative incidence of relapse for patients who achieved CR/CRi.

Univariable analysis of factors associated with achievement of remission is shown in Figure 2A. Normal karyotype, *IDH2*, *NPM1*, or *STAG2* mutations were associated with the achievement of CR/CRi, whereas increased baseline WCC, clinical secondary disease, complex karyotype, +8, −7/abn7q, *MECOM* rearrangement (*MECOM*r), *TP53* mutations, and *JAK2* mutations were associated with higher odds of nonresponse. On multivariable analysis, *IDH2* and *STAG2* remained associated with CR/CRi, with nonresponse associated with clinical secondary disease, +8, and *MECOM*r (supplemental Table 3). Response rate in these groups and other variables of interest are shown in Figure 2B.

In patients achieving CR/CRi, the cumulative incidence of relapse was 14% at 6 months and 33% at 12 months (supplemental Figure 2C). Of a total of 146 relapses, there were 6 patients with measurable residual disease (MRD) relapse only.

### Survival outcomes with venetoclax and azacitidine

**Figure 3.**
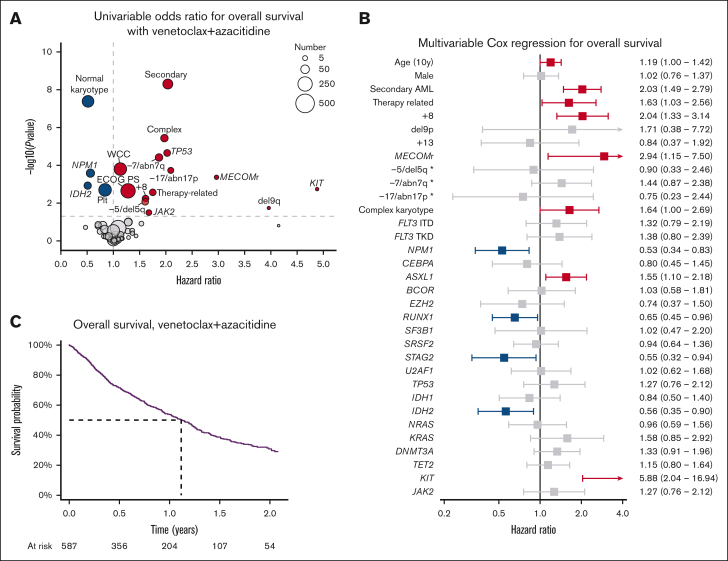
**Characteristics associated with OS with venetoclax and azacitidine.** (A) Univariable hazard ratios for OS. (B) Multivariable Cox regression for OS. (C) Kaplan-Meier plot of OS. ∗Cytogenetic change in absence of complex karyotype.

Day 30 and day 60 mortality were 5% and 8%, respectively. The median OS was 13.6 months (95% confidence interval [CI], 11.7-15.1), with 54% of patients surviving to 12 months and 39% to 18 months (Figure 3A). Survival was not associated with the number of patients treated at each center (supplemental Figure 4). Patients with hematological relapse had poor outcomes, with a median survival after relapse of 1.9 months (supplemental Figure 5).

By univariable Cox regression, normal karyotype, higher baseline platelet count, *NPM1* mutation, and *IDH2* mutation were associated with improved survival (Figure 3B). Worse survival was associated with higher performance status, baseline WCCs, clinical secondary disease, +8, −5/del5q, −7/abn7q, −17/abn17p, *MECOM*r, del9q, and mutations in *JAK2*, *KIT*, or *TP53.* Multivariable regression including factors significant on univariable analysis and genomic abnormalities present in >3% of patients is shown in Figure 3C. Mutations in *NPM1, RUNX1, STAG2*, and *IDH2* were associated with improved survival, whereas age, secondary and therapy-related AML, +8, inv3/t(3;3), complex karyotype, *ASXL1*, and *KIT* mutations were associated with poorer survival.

Patients who had received prior active therapy had a numerically lower CR/CRi rate than patients with previously untreated MDS or MDS/myeloproliferative neoplasm (MPN, 47% vs 58%) but a similar median survival (8.9 vs 9.3 months), noting that this analysis is limited by the small number of patients with prior therapy (n = 15).

### Outcomes in WHO and ICC defined classes with venetoclax and azacitidine

Rates of CR/CRi and survival varied by WHO and ICC classification groups, with the best outcomes in AML with *NPM1* mutations (CR/CRi, 78%; median OS, 22 months) and the poorest with *MECOM*r (CR/CRi, 17%; median OS, 4 months) and *TP53* mutations (CR/CRi, 49%; median OS, 8 months; supplemental Figure 6A,C). Within the MDS-related category for both classifications, those with MDS-related mutations had better outcomes than those with MDS-related cytogenetics (supplemental Figure 6B).

### Prognostic utility of MRC 2010, ELN 2022 and proposed risk scores

**Figure 4.**
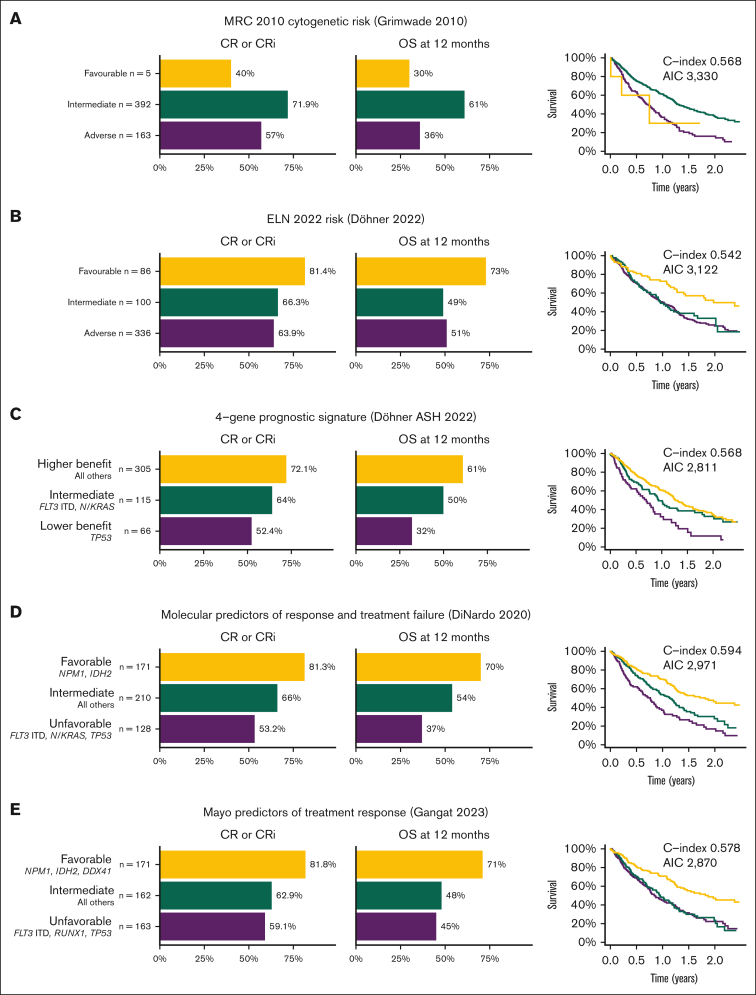
**Rates of CR/CRi, 12-month OS, and Kaplan-Meier survival plots for established and proposed AML prognostication systems for patients treated with venetoclax and azacitidine.** (A) MRC 2010 cytogenetic risk.^9^ (B) ELN 2022 risk.^22^ (C) 4-gene prognostic signature proposed by Döhner et al, from VIALE-A study.^7^ (D) Molecular predictors of response and treatment failure, DiNardo et al.^23^ (E) Mayo predictors of treatment response, Gangat et al.^10^

Most AML prognostic systems have been derived from cohorts of patients treated with intensive chemotherapy, and these may not predict outcomes in patients treated with venetoclax-based nonintensive regimens. We analyzed the outcomes of patients based on previously established and recently proposed prognostic systems. Using the MRC cytogenetic risk,^9^ intermediate and adverse cytogenetics were clearly separable, with small numbers in the favorable risk group precluding any conclusions. The ELN 2022^22^ favorable category had superior outcomes, whereas the intermediate and adverse groups were very similar. Prognostic systems derived from cohorts treated with venetoclax and azacitidine, such as those proposed by Döhner et al,^7^ DiNardo et al,^23^ and Gangat et al,^10^ had better discrimination between the 3 groups (Figure 4), although C-index was <0.6 for all models, suggesting that further improvements are required.

### Outcomes with venetoclax and LDAC

Sixty-seven patients were treated with venetoclax and LDAC, with most having a normal karyotype (72%) and *NPM1* mutation (56%). Seventy-three percent of patients achieved CR/CRi (Table 2), which was higher in those with de novo disease (82%), normal karyotype (78%), and with mutations in *NPM1* (81%) or *IDH2* (92%) and lower with secondary (56%) or therapy-related disease (40%) or +8 (20%) (supplemental Figure 7). The median OS was 10.9 months (95% CI, 8.8- 20.2), with 12-month OS 46% and 18-month OS 31% (supplemental Figure 8).

### Outcomes in patients with *NPM1* mutated AML

In patients with *NPM1* mutated (*NPM1*^mut^) AML receiving either azacitidine or LDAC, CR/CRi was achieved in 78%, 18-month OS was 50%, and the median OS was 17.2 months (supplemental Table 4). Those with a *FLT3*-ITD comutation had worse outcomes, with CR/CRi of 67% vs 82% (*P* = .05), a median OS of 12 months vs not reached, and 18-month OS of 30% vs 56% (*P* = .01; supplemental Figure 9A). Patients with both FLT3-ITD and DNMT3A mutations had the poorest outcomes, with a median OS of 7.4 months (supplemental Figure 9B).

Of the 149 patients with *NPM1* mutated AML, 112 received venetoclax with azacitidine and 37 with LDAC, with no identifiable difference in patient characteristics (supplemental Table 3). Similar outcomes were achieved with the 2 regimens, with CR/CRi of 78% with azacitidine and 81% with LDAC (*P* = .7), a median OS of 22 months vs 17 months, and 18-month OS of 51% vs 47% (*P* = .6) with azacitidine and LDAC, respectively (supplemental Table 3; supplemental Figure 9C).

## Discussion

Here, we describe outcomes of patients with newly diagnosed AML treated with venetoclax and azacitidine or LDAC in, to our knowledge, the largest real-world cohort reported to date, including 53 hospitals across the UK NHS. Response rates and survival were similar to those seen in the randomized studies of venetoclax-azacitidine^2^^,^^24^ and venetoclax-LDAC,^3^^,^^25^ despite the majority of patients being treated during the COVID-19 pandemic. We also report detailed information on blood count recovery and supportive care requirements, providing a useful benchmark for clinicians using these therapies and for studies examining modified dosing regimens.

In the VIALE-A study, the 286 patients receiving venetoclax and azacitidine had a CR/CRi rate of 66.4%, 30-day mortality of 7%, and a median OS of 14.7 months.^2^^,^^24^ The characteristics of patients in our cohort are similar to those in VIALE-A, except for a slightly younger median age (73 vs 76 years) and fewer de novo AML (59% vs 75%), likely due to the exclusion of patients previously treated with hypomethylating therapy in VIALE-A. The outcomes in our cohort mirror those in VIALE-A very closely, with CR/CRi achieved in 67%, a 30-day mortality of 5%, and a median OS of 13.6 months. It has been suggested that real-world outcomes may be poorer than those seen in clinical trials. A single-center comparison of patients receiving venetoclax-azacitidine on and off trial found that those treated off trial had worse OS despite a younger median age, and a systematic review of real-world studies confirmed lower OS than that of VIALE-A.^11^^,^^26^ The comparable results we report here may reflect subjectivity in determining eligibility for intensive chemotherapy and increasing use of venetoclax-azacitidine in patients who may previously have been considered eligible for intensive treatment. This reflects increasing use worldwide of venetoclax-azacitidine in younger and fitter patients, as demonstrated in a systematic review of real-world studies in which the median cohort age was <75 years in 12 of 18 studies.^26^ Supporting this hypothesis are the findings from a recent analysis from 8 US centers that only included patients aged ≥75 years, in which response rates were similar to VIALE-A but survival was worse.^19^

Prognosis in patients with AML is generally assigned using the MRC cytogenetic and/or ELN 2022 risk classifications, both of which were derived from cohorts of patients treated with intensive chemotherapy.^9^^,^^22^ It has become clear that these do not adequately discriminate prognosis in patients receiving venetoclax-based therapies, with a pressing need for new risk-stratification schemes.^7^^,^^8^ A number have been proposed, most of which use mutations only.^7^^,^^10^^,^^12^^,^^23^ Although there are differences between them, recurring themes are seen, with mutations in *NPM1* and *IDH2* being favorable and *FLT3-*ITD, *TP53*, and *N/KRAS* adverse. We demonstrate that these proposed systems perform better than the MRC and ELN risk classifications, but their prognostic power remains limited, and further refinement is necessary. We suggest that a more definitive, unified prognostic classification combining cytogenetic, molecular, and possibly clinical characteristics will require a collaborative international effort to integrate all available clinical trial and real-world data sets.

A number of unexpected associations were noted in our multivariable analysis of clinical, disease, and genomic factors associated with OS. Trisomy 8 was strongly associated with worse outcomes, despite being considered an intermediate-risk cytogenetic abnormality. A similar finding was seen in a recent analysis of patients with high-risk MDS treated with venetoclax and HMA,^27^ and indeed in the 2010 analysis by the MRC, there was a significantly increased risk of death in patients with +8 on multivariable analysis (hazard ratio [HR], 1.33; 95% CI, 1.12-1.57).^9^ We did not find a significant association of *TP53* mutations with survival in multivariable analysis, likely due to their close association with complex karyotype. *FLT3*-ITD was also not prognostic, consistent with data from VIALE-A.^28^ Finally, *ASXL1* mutations were associated with poor outcomes in contrast to *RUNX1* and *STAG2* mutations in which better survival was seen. This further emphasizes the need for dedicated prognostic systems for these patients, given all of these mutations would be considered MDS associated and confer adverse prognosis in the ELN system.

*NPM1*^mut^ AML appears to be uniquely sensitive to venetoclax-based therapies, regardless of the chemotherapy regimen with which it is combined.^23^^,^^29^, ^30^, ^31^ In VIALE-A, the addition of venetoclax increased CR/CRi from 24% to 67% in *NPM1*^mut^ AML, and 10 of the 27 patients were still alive at 2 years,^2^^,^^24^ whereas in VIALE-C, the CR/CRi rate improved from 57% to 79%, and the median OS was 25.3 months.^3^^,^^25^ This study includes, to our knowledge, the largest reported cohort of patients with *NPM1*^mut^ AML receiving venetoclax and demonstrates that outcomes are similar between patients receiving LDAC and azacitidine. We also confirm the detrimental impact of a *FLT3*-ITD comutation within the *NPM1*^mut^ subgroup, despite no impact on the overall population. Longer follow-up will be required to establish whether patients with the genotype *NPM1*^mut^, *FLT3*-ITD negative may be a population in which low-intensity venetoclax-based strategies can result in functional cure, especially in those who achieve MRD-negative remissions.^20^

We recognize a number of limitations to this analysis. Patients were identified, and data were collected retrospectively, which introduces potential selection bias. We attempted to minimize this by identifying and including all patients at each center, using pharmacy records and departmental databases. Given the large number of centers and clinicians involved, it is possible there was variability in collection of data, in particular for assigning response and other potentially subjective end points. Treatment and supportive care data were only available for a subgroup of patients, which introduces another potential source of reporting bias. The large number of patients collected and our focus on OS as the main end point (rather than event-free or relapse-free survival that are more susceptible to clinician judgment and missing data) increase the likelihood that our results are robust.

Conflict-of-interest disclosure: J. Othman declares honoraria from Astellas and Jazz Pharmaceuticals; and speaker’s fees and advisory board fees from Pfizer, Jazz Pharmaceuticals, Astellas, and AbbVie. P.G. declares honoraria from Astellas. F.H. declares meeting sponsorship and honoraria from AbbVie. R. Dang declares meeting sponsorship from Jazz; and honoraria from AbbVie. J.V. declares meeting support from BeiGene, Janssen, and Jazz; and honoraria from AbbVie and AstraZeneca. P. Krishnamurthy declares honoraria from Jazz, Astellas, and Gilead; speaker’s bureau with Astellas; and consultancy for Jazz and Gilead. A.-L.L. declares honoraria from Astella, AbbVie, Amgen, Kite, Novartis, Jazz, and Daiichi Sankyo; and speaker’s bureau with Kite, Takeda, and Astellas. V.M. declares consultancy and honoraria from AbbVie. N.F. declares investigator meetings with Novartis and MEI Pharma. A.K. declares meeting sponsorship from Jazz, Medac, and Servier; speaker’s bureau with AbbVie, Astellas, Jazz and Servier; and consultancy/advisory board for TC BioPharm, Novartis, Synairgen, and Takeda. J. O'Nions declares honoraria from AbbVie, Astellas, Janssen, Jazz and Servier. R. Dillon declares research funding from AbbVie and Amgen; and consultancy with Astellas, Pfizer, Novartis, Jazz, BeiGene, Shattuck, and AvenCell. The remaining authors declare no competing financial interests.
