# Supplementary material for: Real-world outcomes of newly diagnosed AML treated with venetoclax and azacitidine or low-dose cytarabine in the UK NHS
Source: Blood Neoplasia. 2024 May 23;1(3):100017. doi: 10.1016/j.bneo.2024.100017 (PMC12082122; doi:10.1016/j.bneo.2024.100017)

Real-world outcomes of newly diagnosed AML treated with venetoclax and azacitidine or low-dose cytarabine in the UK National Health Service

Supplemental appendix

Jad Othman[1,2,3], Ho Pui Jeff Lam[4], Sarah Leong[5], Faisal Basheer[6], Islam Abdallah[7], Kathryn Fleming[8], Priyanka Mehta[8], Heba Yassin[9], John Laurie[9], Michael Austin[10], Paolo Gallipoli[10], Thomas Taylor[11], Mike Dennis[12], Johnathon Elliot[12], Georgina Clarke[13], Raymond Dang[13], Jennifer Vidler[14], Pramila Krishnamurthy[14], Anne-Louise Latif[15], Pallavi Kalkur[16], Maryam Shahidianakbar[17], Victoria Campbell[18], Deepak Mannari[19], Emily Sutherland[20], Thishakya Wickramaratne[21], Angela Collins[22], Rui Zhao[23], Herng Mak[24], Edward Belsham[25], Shabnam Banerjee[26], Jamila Bashir[27], Srinivas Pillai[28], Richard Whitmill[29], Sofia Galli[30], Mariam Amer[31], Vidhya Murthy[32], Duncan Murray[33], Farooq Wandroo[34], Francesca Hogan[35], Francesca Crolla[36], Nicole Fowler[37], Anjum Khan[7], Jenny O'Nions[4], Richard Dillon[1,2]

[1]Department of Medical and Molecular Genetics, King's College London, [2]Guy's and St Thomas' NHS Foundation Trust, [3]Faculty of Medicine and Health, [4]University College London Hospital NHS Foundation Trust, London, United Kingdom, [5]University College London Hospital NHS Foundation Trust, London, United Kingdom, University of Sydney, [6]Addenbrooke's Hospital, Cambridge, United Kingdom, [7]Department of Haematology, Leeds Teaching Hospitals Trust, Leeds, United Kingdom, [8]University Hospital Bristol, Bristol, United Kingdom, [9]University Hospitals Sussex NHS Foundation Trust, Worthing, United Kingdom, [10]Barts Cancer Institute, Queen Mary University of London, London, United Kingdom;, [11]Nottingham University Hospital, Nottingham, United Kingdom, [12]The Christie NHS Foundation Trust, Manchester, United Kingdom, [13]James Cook University Hospital, Middlesbrough, United Kingdom, [14]King's College Hospital, London, United Kingdom, [15]Department of Haematology, Queen Elizabeth University Hospital, Glasgow, United Kingdom, [16]Southend University Hospital, Southend-on-sea, United Kingdom, [17]Mersey and West Lancashire Teaching Hospitals NHS trust, Whiston, United Kingdom, [18]Western General Hospital, NHS Lothian, Edinburgh, [19]Musgrove Park Hospital, Somerset NHS Foundation Trust, Taunton, [20]City Hospitals Sunderland NHS Trust, Sunderland, United Kingdom, [21]Blackpool Teaching Hospitals NHS Foundation Trust, [22]Norfolk and Norwich University Hospitals NHS Foundation Trust, Norwich, [23]Torbay Hosital, Torquay, United Kingdom, [24]University Hospitals Sussex NHS Foundation Trust, Brighton, United Kingdom, [25]Portsmouth Hospitals University NHS Trust, [26]Queens Hospital, Barking, Havering and Redbridge University Hospitals NHS Trust, [27]University Hospitals of Derby and Burton NHS Foundation Trust, [28]Royal Stoke University Hospital, University Hospital of North Midlands NHS Trust, Stoke-on-Trent, United Kingdom, [29]New Cross Hospital, The Royal Wolverhampton NHS Trust, Wolverhampton, [30]Frimley Park Hospital, London, United Kingdom, [31]University Hospital Southampton, Southampton, United Kingdom, [32]Centre for Clinical Haematology, University Hospitals Birmingham, Birmingham, United Kingdom, [33]University Hospitals Coventry and Warwickshire NHS Trust, Coventry, [34]Sandwell and West Birmingham Hospitals NHS Trust, Birmingham, [35]University Hospital of Wales, Cardiff, United Kingdom, [36]University Hospitals Plymouth NHS Trust, [37]Royal Cornwall Hospitals NHS Trust

Table of Contents

List of centres and contributing clinicians 3

Supplemental methods 4

Table S1 – therapy administered and toxicity in cycles 1 to 4 5

Table S2 – blood count recovery in cycles 1 to 4 6

Table S3 – multivariable analysis of factors associated with not achieving CR/CRi 7

Table S4 – characteristics and outcomes of patients with *NPM1* mutated AML 8

Figure S1 – number of patients included from each participating site 9

Figure S2 – density plots of number of days of venetoclax, hospitalisation and transfusion in cycles 1 to 4 10

Figure S3 – overall survival by best response with venetoclax and azacitidine 11

Figure S4 – overall survival by number of patients treated at site (venetoclax and azacitidine) 12

Figure S5 – overall survival from haematological relapse after venetoclax and azacitidine 13

Figure S6 – outcomes by WHO and ICC defined AML classification (venetoclax and azacitidine) 14

Figure S7 – response rates in patients treated with venetoclax and low-dose cytarabine 15

Figure S8 – overall survival for venetoclax and low-dose cytarabine 16

Figure S9 – overall survival in *NPM1* mutated AML 17

List of centres and contributing clinicians

Barts Cancer Institute - Paolo Gallipoli, Michael Austin, Kayleigh McCloskey, Matthew Smith, Michael Hamblin; Royal United Hospital Bath - Sally Moore, Laura Anderson; Queen Elizabeth Hospital Birmingham - Charles Craddock, Justin Loke; Birmingham Heartlands Hospital - Vidhya Murthy; Blackpool Hospital - Seye Kolade, Thishakya Wickramaratne, Asra Khan, Usman Afzal; Royal Sussex County Hospital - Tom Rider, Herng Mak; Bristol Royal Infirmary - Priyanka Mehta, Kathryn Fleming, Joe Cross; Addenbrooke's Hospital - Faisal Basheer, Charles Crawley, Shahzad Orthi, Jayalal Jayalal, Lee Mynott; University Hospital of Wales - Steven Knapper, Francesca Hogan, Victoria Ware; The Christie Hospital - Mike Dennis, Johnathan Elliot; University Hospital Coventry & Warwickshire - Duncan Murray, Beth Harrison, Martin Dyson, Jacob Thanakamma; Royal Derby Hospital - Ian Amott, Jamila Bashir; Kent and Canterbury Hospital - Sreetharan Munisamy; Frimley Park Hospital - Sofia Galli; Gloucestershire Royal Hospital - Adam Rye; Great Western Hospital - Alex Sternberg; Guy’s Hospital - Richard Dillon, Jad Othman, Jamie Saunders, Ruebina Amofa; Basingstoke and North Hampshire Hospital - Henna Wong, Sylwia Simpson, Katherine Smith, Saniya Dhawan; Hull and East Yorkshire Hospitals - Simone Green, Mayanka Narayanan; Hammersmith Hospital - Renuka Palanicawandar; Ipswich Hospital - Mahesh Prahladan, Ioana Whalley; James Cook University Hospital - Raymond Dang, Oghenemaro Okah Avae, Georgina Clarke; King’s College Hospital - Pramila Krishnamurthy, Jennifer Vidler; Kingston Hospital - Stefania Bonetto; St James' Cancer Institute - Anjum Khan, Manish Jain, Islam Abdallah, Chun Huat Teh, Richard Kelly; University Hospitals of Leicester - Katherine Hodgson, Alex Bashford; Lincoln County Hospital - Charlotte Kallmeyer; Norfolk and Norwich University Hospital - Angela Collins; University Hospitals of North Midlands - Srinivas Pillai; Peterborough City Hospital - Sateesh Nagumantry; Northwick Park Hospital - Vaitsa Katsomitrou, Hasan Jamjoom; Nottingham University Hospitals - Jennifer Byrne, Thomas Taylor, Denise Badder; Royal Oldham Hospital - David Osborne, Odong Ochaya; Derriford Hospital - Patrick Medd, Francesca Crolla; Queen Alexandra Hospital, Portsmouth - Edward Belsham, Behnaz Mobashwera; Queen Elizabeth University Hospital - Anne-Louise Latif, Cara Manson; Queen’s Hospital Romford - Abbas Zaidi, Shabnam Banerjee, Jesca Boot; Royal Cornwall Hospital - David Tucker, Nicole Fowler, Claudia Bedford; Royal Devon and Exeter Hospital - Tom Coats; Royal Marsden Hospital - David Taussig, Madhu Sivarajah; Royal Surrey County Hospital - Elisabeth Grey-Davies; Royal Wolverhampton Hospital - Richard Whitmill; Royal Hallamshire Hospital - Alex Kanellopoulos; Royal Shrewsbury Hospital - George Cherian, Sarah Lane; Musgrove Park Hospital - Deepak Mannari, Francesca Crolla, Jayne Foot, Elizabeth Firth; Sunderland Royal Hospital - Scott Marshall, Emily Sutherland; University Hospital Southampton - Chris Dalley, Mariam Amer; Southend University Hospital - Pallavi Kalkur; Whiston Hospital - Eleana Loizou, Maryam Shahidianakbar, Emma Livesey, Dominika Radzova; Sandwell General Hospital - Farooq Wandroo; Torbay Hospital - Rui Zhao; University College Hospital - Jenny O'nions, Ho Pui Jeff Lam, Sarah Leong, Asim Khwaja; Western General Hospital - Victoria Campbell; Worthing Hospital - John Laurie, Heba Yassin

Supplemental methods

Guidance provided to clinicians on the use of venetoclax as an emergency measure during the COVID pandemic (version 4, dated 16/6/2020)

Venetoclax (VEN) based treatment protocols may lower treatment-related toxicity compared to intensive therapy. Treatment is largely delivered as an outpatient. Remission rates (CR+CRi) parallel those achieved with intensive therapy in older patients. The use of VEN based regimens is therefore attractive both to reduce pressure on the NHS and protect patients at especially high risk during the COVID19 outbreak.

Long-term follow up data are immature and in general the approach we recommend is to use VEN to bridge patients through the COVID19 epidemic with a view to delivering definitive therapy possibly including transplant later on. Decisions regarding subsequent treatment should be made on a case by case basis and MRD status will be particularly informative in this regard.

Venetoclax has been approved by NHS England for first line treatment during the epidemic, azacytidine is funded for use in conjunction. Based on the limited information available, we recommend the azacytidine schedule for most patients. However, VEN+LDAC appears equally effective for patients with *NPM1* or *IDH1/2* mutations and may be preferred for other patients on practical grounds.

- Any non-CBF patient aged >60y
- Patients with an *NPM1* or *IDH1/2* mutation aged >50y or with comorbidities
- Patients with the *NPM1*^{mut} *FLT3* ITD^{neg} genotype of any age

The following treatment schedule is recommended (other schedules may be used according to established local practice)

<u>Azacitidine schedule:</u>

- Azacitidine 75mg/m² SC, once a day D1-7 (or D1-5 and D8-9)
- Venetoclax (cycle 1) 100mg D1, 200mg D2, 300mg D3 and **100mg*** D4-D28 orally once daily
  please note the dose drops on D4 to account for the azole loading
  (cycle 2 onwards) 100mg D1-D28 orally (see below for guidance on changing number of days per cycle)
- Posaconazole (cycle 1) 300mg twice daily on **D4** and once daily on **D5-28**
  (cycle 2 onwards) 300mg once daily on D1-D28

  or
- Voriconazole (cycle 1) 400mg twice daily on **D4** and once daily on **D5-28**
  (cycle 2 onwards) 200mg twice daily on D1-D28

<u>Cytarabine schedule:</u>

- Cytarabine 20mg/m2 SC once a day on D1 to 10
- Venetoclax (cycle 1) 100mg D1, 200mg D2, 300mg D3 and **100mg*** D4-D28 orally once daily
  please note the dose drops on D4 to account for the azole loading
  (cycle 2 onwards) 100mg D1-D28 orally (see below for guidance on changing number of days per cycle)
- Posaconazole (cycle 1) 300mg twice daily on **D4** and once daily on **D5-28**
  (cycle 2 onwards) 300mg once daily on D1-D28

  or
- Voriconazole (cycle 1) 400mg twice daily on **D4** and once daily on **D5-28**
  (cycle 2 onwards) 200mg twice daily on D1-D28

Note: this recommendation was produced in a very short time frame at the beginning of the coronavirus pandemic and the discrepancy in the day 3 dose (300mg) compared to the recommendation in the venetoclax product information (400mg) was not noted. Once venetoclax was approved by NICE the recommended dose titration in the Summary of Product Characteristics (SmPC) was 100mg, 200mg and 400mg on days 1, 2 and 3.

Table S1 – therapy administered and toxicity in cycles 1 to 4

	**Cycle 1**	**Cycle 2**	**Cycle 3**	**Cycle 4**
Venetoclax dose				
50 mg	12 (2.2%)	14 (4.3%)	14 (5.9%)	9 (4.7%)
70 mg	3 (0.6%)	2 (0.6%)	3 (1.3%)	2 (1.0%)
100 mg	515 (96%)	306 (95%)	220 (92%)	180 (94%)
120 mg			1 (0.4%)	
200 mg	2 (0.4%)			
300 mg	2 (0.4%)	1 (0.3%)		
400 mg	3 (0.6%)		1 (0.4%)	1 (0.5%)
Median number of days of venetoclax (IQR)	28 (24, 28)	28 (21, 28)	21 (14, 28)	21 (14, 28)
Azole antifungal				
Posaconazole	464 (86%)	275 (85%)	203 (85%)	160 (84%)
Voriconazole	67 (12%)	44 (14%)	34 (14%)	29 (15%)
Isavuconazole	1 (0.2%)	1 (0.3%)		
Fluconazole		1 (0.3%)		
None	5 (0.9%)	1 (0.3%)	2 (0.8%)	2 (1.0%)
Median days between day 1 and next cycle (IQR)	41 (35, 49)	40 (29, 49)	35 (28, 49)	
Proportion with any hospital admission	93%	37%	18%	14%
Median days in hospital (IQR)	14 (8, 27)	0 (0, 7)	0 (0, 0)	0 (0, 0)
Proportion with any ICU admission	4.0%	0.3%	0.5%	0.6%
Median days in ICU (IQR)	0 (0, 0)	0 (0, 0)	0 (0, 0)	0 (0, 0)
Required IV antibiotics	63%	23%	13%	9.5%
Median days on IV antibiotics (IQR)	5 (0, 12)	0 (0, 0)	0 (0, 0)	0 (0, 0)
Required red blood cell transfusion	85%	47%	31%	23%
Median number RBC units transfused (IQR)	5 (2, 8)	0 (0, 3)	0 (0, 1)	0 (0, 0)
Required platelet transfusion	59%	27%	19%	19%
Median number platelet units transfused (IQR)	1 (0, 5)	0 (0, 1)	0 (0, 0)	0 (0, 0)

Table S2 – blood count recovery in cycles 1 to 4

Neutrophil recovery

**Including all patients**						
	**All patients**		**Azacitidine**		**Low-dose cytarabine**	
	**Median days (95% CI)**	**Recovered by day 28**	**Median days (95% CI)**	**Recovered by day 28**	**Median days (95% CI)**	**Recovered by day 28**
Cycle 1	33 (32, 35)	33%	34 (32, 35)	30%	26 (23, 33)	56%
Cycle 2	25 (20, 28)	56%	26 (21, 30)	55%	15 (0, 31)	68%
Cycle 3	19 (14, 22)	67%	19 (14, 22)	68%	22 (0, 35)	60%
Cycle 4	7 (1, 13)	74%	7 (2, 13)	73%	0 (0, 29)	79%
**Only patients with CR/CRi**						
	**All patients**		**Azacitidine**		**Low-dose cytarabine**	
	**Median days (95% CI)**	**Recovered by day 28**	**Median days (95% CI)**	**Recovered by day 28**	**Median days (95% CI)**	**Recovered by day 28**
Cycle 1	32 (30, 33)	37%	32 (30, 34)	35%	27 (24, 33)	57%
Cycle 2	21 (12, 26)	61%	21 (12, 28)	59%	11 (0, 31)	70%
Cycle 3	19 (13, 22)	69%	17 (12, 22)	70%	22 (0, 35)	58%
Cycle 4	7 (1, 13)	74%	7 (3, 14)	73%	0 (0, 28)	83%

Platelet recovery

**Including all patients**						
	**All patients**		**Azacitidine**		**Low-dose cytarabine**	
	**Median days (95% CI)**	**Recovered by day 28**	**Median days (95% CI)**	**Recovered by day 28**	**Median days (95% CI)**	**Recovered by day 28**
Cycle 1	21 (20, 24)	67%	21 (20, 24)	68%	23 (20, 32)	65%
Cycle 2	0 (0, 5)	72%	0 (0, 4)	73%	19 (0, 50)	61%
Cycle 3	0 (0, 0)	76%	0 (0, 0)	77%	0 (0, 40)	68%
Cycle 4	0 (0, 0)	81%	0 (0, 0)	80%	0 (0, 42)	84%
**Only patients with CR/CRi**						
	**All patients**		**Azacitidine**		**Low-dose cytarabine**	
	**Median days (95% CI)**	**Recovered by day 28**	**Median days (95% CI)**	**Recovered by day 28**	**Median days (95% CI)**	**Recovered by day 28**
Cycle 1	20 (18, 21)	75%	20 (17, 21)	76%	21 (20, 27)	74%
Cycle 2	0 (0, 0)	76%	0 (0, 0)	78%	15 (0, 50)	62%
Cycle 3	0 (0, 0)	78%	0 (0, 0)	79%	0 (0, 40)	71%
Cycle 4	0 (0, 0)	82%	0 (0, 0)	81%	0 (0, 15)	89%

Table S3 – multivariable analysis of factors associated with not achieving CR/CRi

Characteristic	Odds ratio	95% CI	p-value
Age	1.06	0.80 - 1.42	0.7
Male	0.89	0.54 - 1.45	0.6
Clinical disease type			
Secondary	**2.15**	**1.28 - 3.63**	**0.004**
Therapy-related	1.48	0.64 - 3.32	0.3
+8	**2.82**	**1.35 - 5.96**	**0.006**
del9q	-	0.00 - NA	>0.9
+13	0.67	0.15 - 2.53	0.6
MECOM rearrangement	**17.9**	**2.29 - 420**	**0.020**
-5/del5q	0.86	0.15 - 3.93	0.9
-7/abn7q	1.33	0.57 - 3.03	0.5
-17/abn17p	1.13	0.10 - 10.5	>0.9
Complex karyotype	1.44	0.63 - 3.23	0.4
FLT3-ITD	2.19	0.93 - 5.04	0.066
FLT3 TKD	0.69	0.22 - 1.84	0.5
NPM1	0.68	0.32 - 1.39	0.3
CEBPA	0.68	0.22 - 1.81	0.5
ASXL1	1.38	0.78 - 2.42	0.3
BCOR	0.55	0.18 - 1.45	0.2
EZH2	1.01	0.30 - 3.20	>0.9
RUNX1	1.15	0.61 - 2.13	0.7
SF3B1	0.47	0.06 - 2.31	0.4
SRSF2	1.10	0.58 - 2.07	0.8
STAG2	**0.22**	**0.07 - 0.60**	**0.006**
U2AF1	0.94	0.39 - 2.18	0.9
TP53	1.40	0.59 - 3.30	0.4
IDH1	0.63	0.26 - 1.43	0.3
IDH2	**0.29**	**0.12 - 0.63**	**0.003**
NRAS	0.78	0.33 - 1.72	0.5
KRAS	1.44	0.47 - 4.27	0.5
DNMT3A	1.07	0.53 - 2.09	0.9
TET2	0.99	0.55 - 1.77	>0.9
KIT	3.05	0.35 - 28.7	0.3
JAK2	2.24	0.93 - 5.51	0.074

Odds ratio >1 indicates higher odds of not achieving remission

Table S4 – characteristics and outcomes of patients with *NPM1* mutated AML

Characteristic	All *NPM1* N = 149	Azacitidine N = 112	LDAC N = 37	p-value
Median age (IQR)	73 (69, 76)	73 (69, 77)	73 (65, 76)	0.2
Female	75 (50%)	55 (49%)	20 (54%)	0.6
Clinical disease type				0.3
De novo	114 (77%)	82 (73%)	32 (86%)	
Secondary	21 (14%)	18 (16%)	3 (8.1%)	
Therapy-related	14 (9.4%)	12 (11%)	2 (5.4%)	
Baseline blood counts				
WCC, median (IQR)	16 (4, 54)	14 (3, 44)	25 (5, 86)	0.2
Haemoglobin, median (IQR)	91 (77, 103)	95 (78, 107)	90 (76, 101)	0.5
Platelet count, median (IQR)	73 (36, 110)	83 (42, 117)	42 (28, 88)	0.033
Missing	53	44	9	
Bone marrow blast %, median (IQR)	65 (30, 84)	60 (30, 83)	65 (38, 84)	>0.9
Missing	*61*	*51*	*10*	
Cytogenetic/FISH abnormalities				
+8	11 (7.7%)	9 (8.5%)	2 (5.6%)	0.7
Complex karyotype	3 (2.1%)	3 (2.8%)	0 (0%)	0.6
Normal karyotype	113 (80%)	82 (77%)	31 (86%)	0.3
FLT3 ITD	36 (24%)	27 (24%)	9 (24%)	>0.9
FLT3 TKD	19 (13%)	15 (14%)	4 (11%)	>0.9
Mutations on NGS panel				
DNMT3A	33 (31%)	24 (30%)	9 (31%)	>0.9
IDH1	12 (11%)	11 (14%)	1 (3.4%)	0.2
IDH2	23 (21%)	15 (19%)	8 (28%)	0.3
TP53	2 (1.9%)	2 (2.5%)	0 (0%)	>0.9
TET2	24 (22%)	16 (20%)	8 (28%)	0.4
Missing	*41*	*33*	*8*	
ELN 2022 risk group				0.6
Favorable	106 (73%)	78 (72%)	28 (78%)	
Intermediate	34 (23%)	26 (24%)	8 (22%)	
Adverse	5 (3.4%)	5 (4.6%)	0 (0%)	
Not able to assign	*4*	*3*	*1*	
**Outcomes**				
CR or CRi	116 (78%)	87 (78%)	29 (81%)	0.7
Median OS (months)	17	22	17	0.6
12 month OS	66%	68%	60%	
18 month OS	50%	51%	47%	

Figure S1 – number of patients included from each participating site

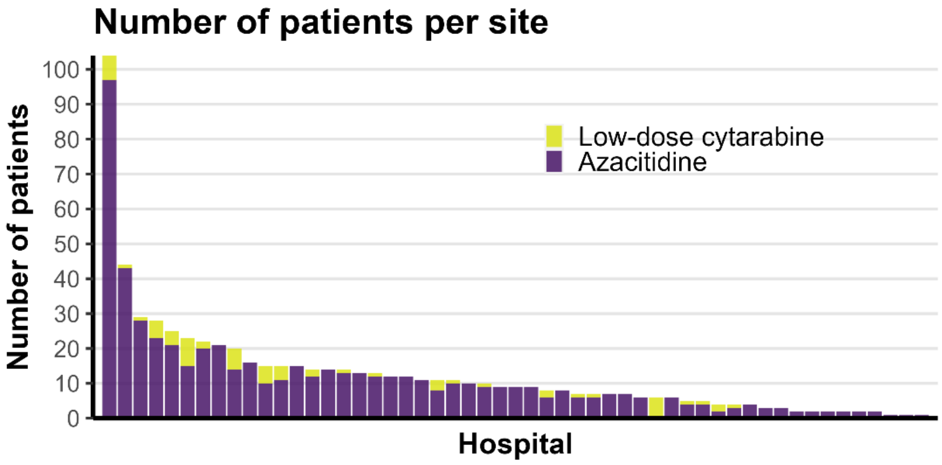

Figure S2 – density plots of number of days of venetoclax, hospitalisation and transfusion in cycles 1 to 4

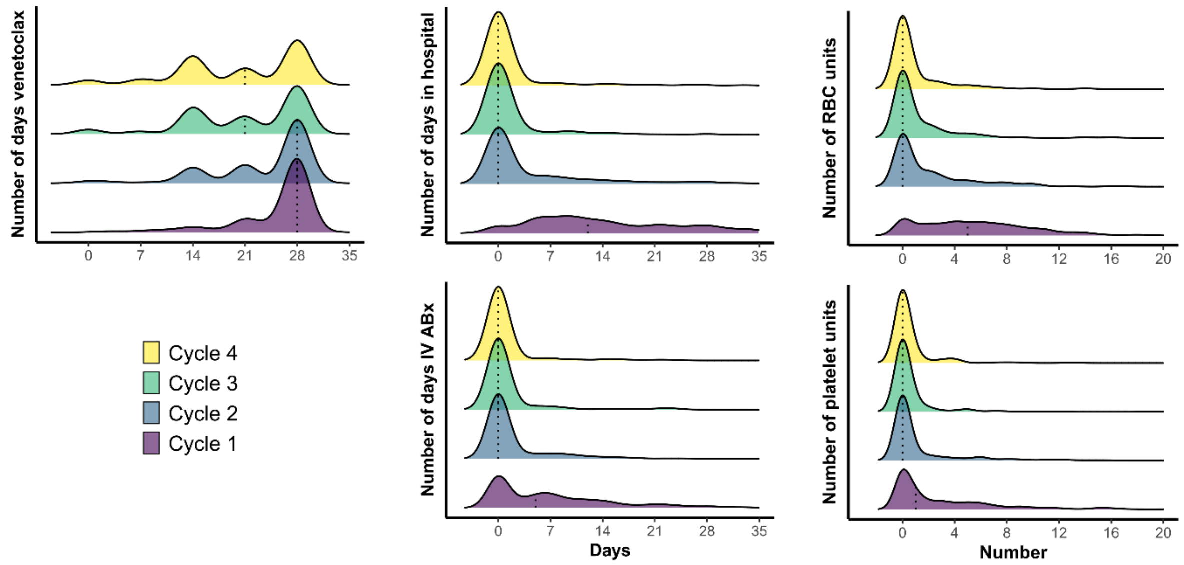

Figure S3 – overall survival by best response with venetoclax and azacitidine

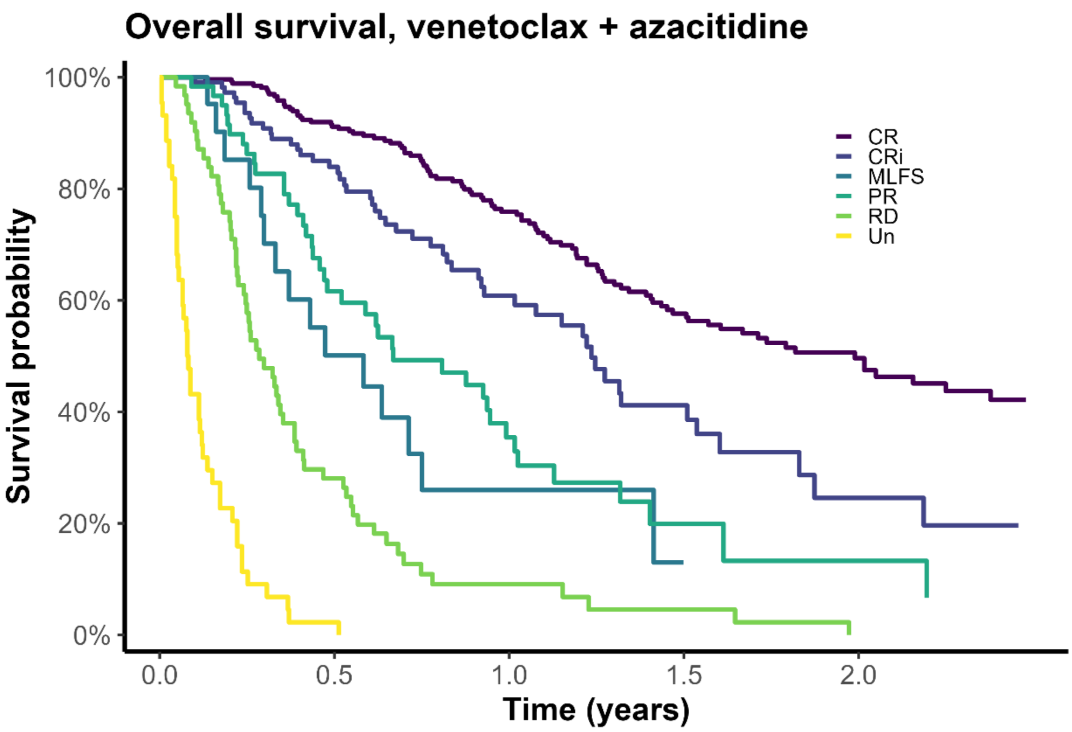

Number at risk

	0.0	0.5	1.0	1.5	2.0
CR	272	222	146	87	48
CRi	113	76	37	16	5
MLFS	21	9	4	0	0
PR	61	30	14	3	2
RD	62	17	4	2	0
Un	44	1	0	0	0

Figure S4 – overall survival by number of patients treated at site (venetoclax and azacitidine)

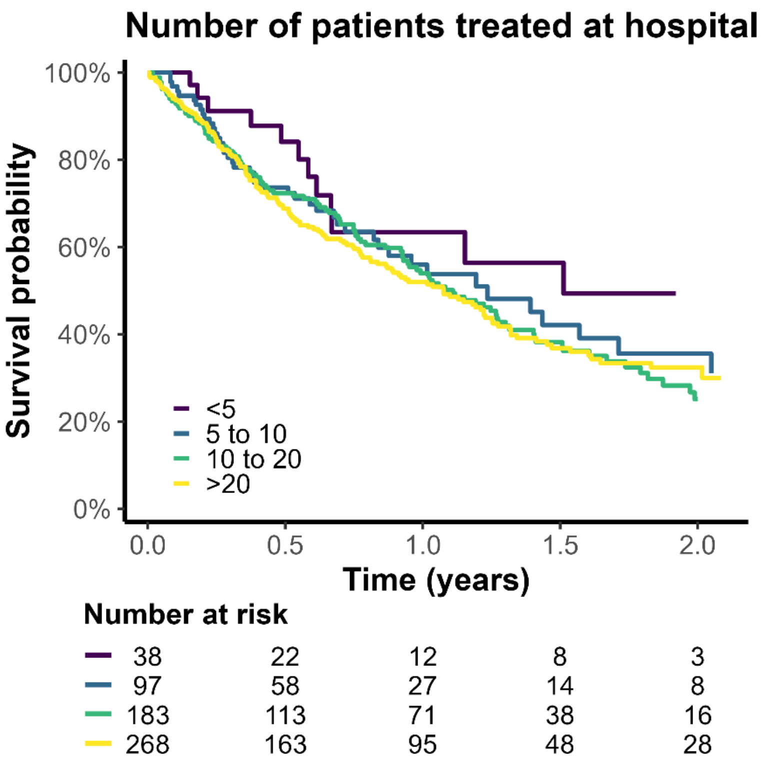

Figure S5 – overall survival from haematological relapse after venetoclax and azacitidine

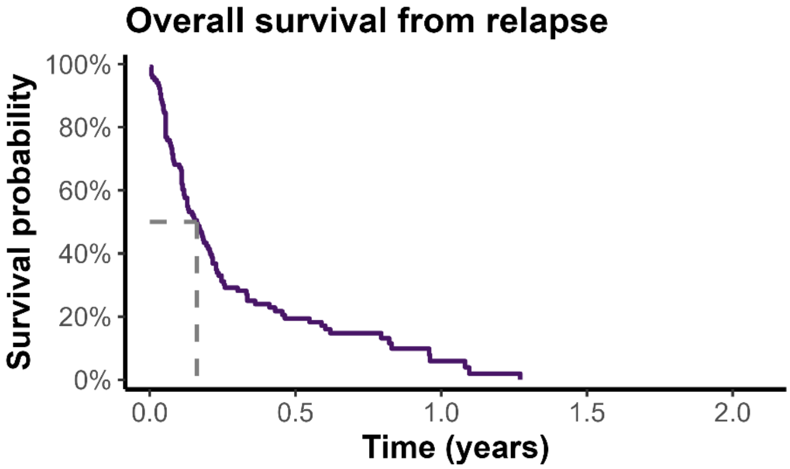

Number at risk	119	17	3	0	0

Figure S6 – outcomes by WHO and ICC defined AML classification (venetoclax and azacitidine)

A. CR/CRi and 12-month OS by WHO/ICC classification

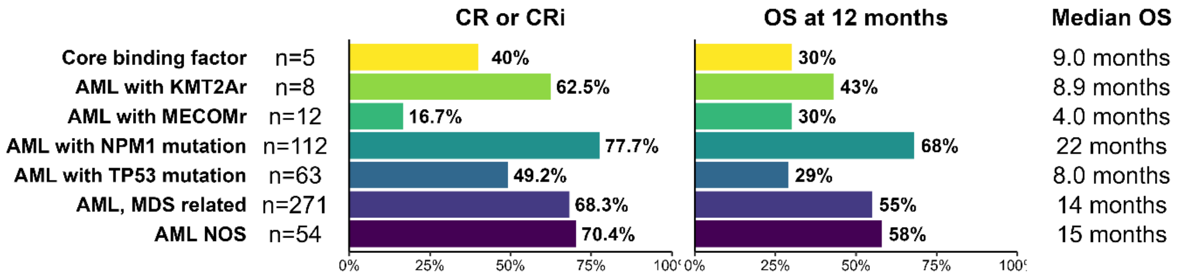

AML, MDS related includes patients meeting criteria by either classification system

B. CR/CRi and 12-month OS by WHO and ICC criteria for MDS-related changes

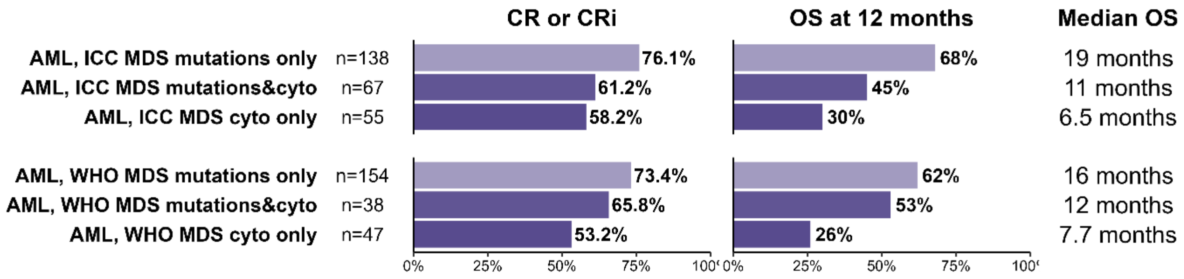

TP53 mutations are excluded from all MDS-related classifications for this analysis

C. Overall survival by by WHO/ICC classification

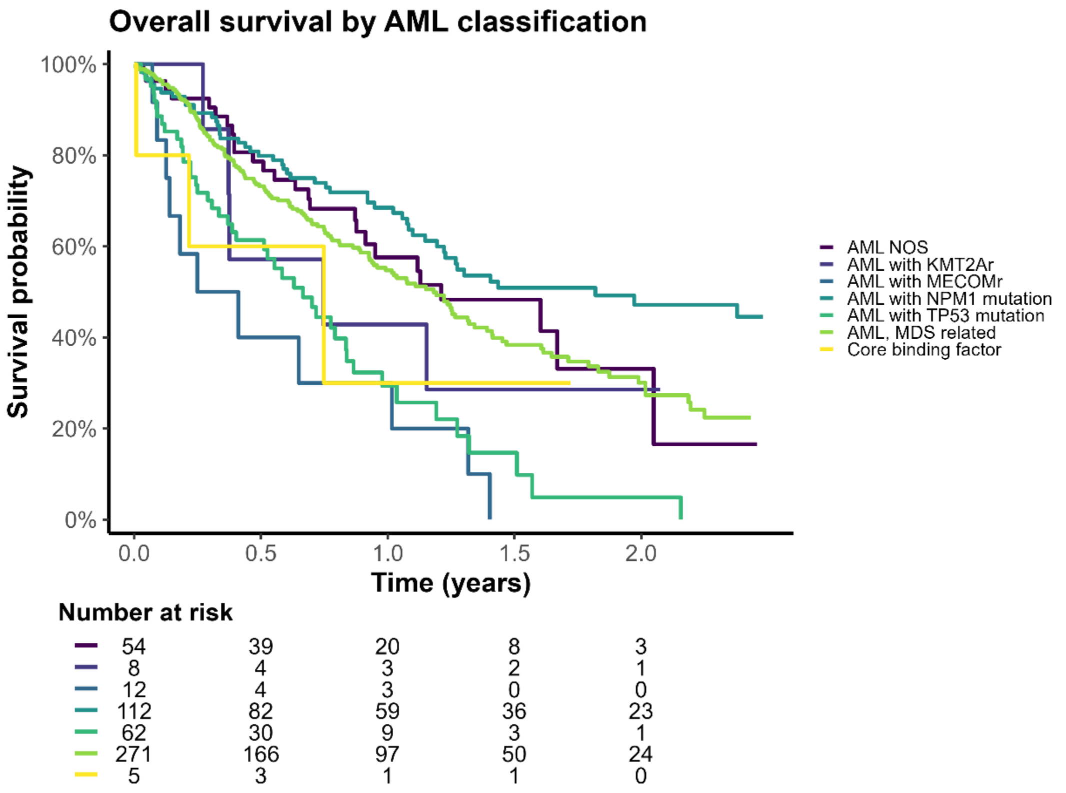

Figure S7 – response rates in patients treated with venetoclax and low-dose cytarabine

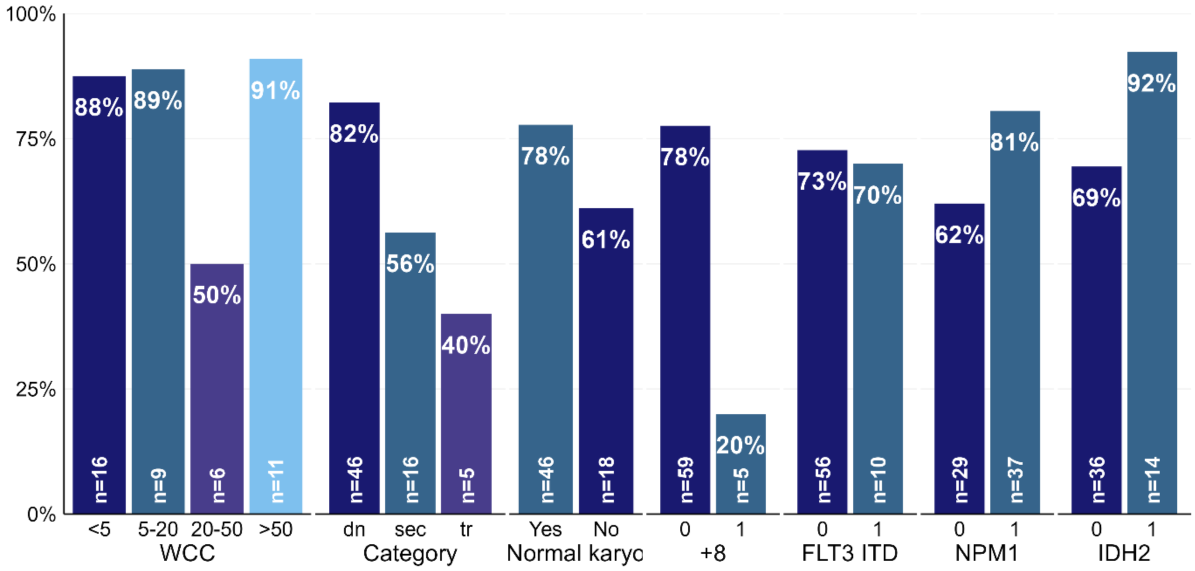

Figure S8 – overall survival for venetoclax and low-dose cytarabine

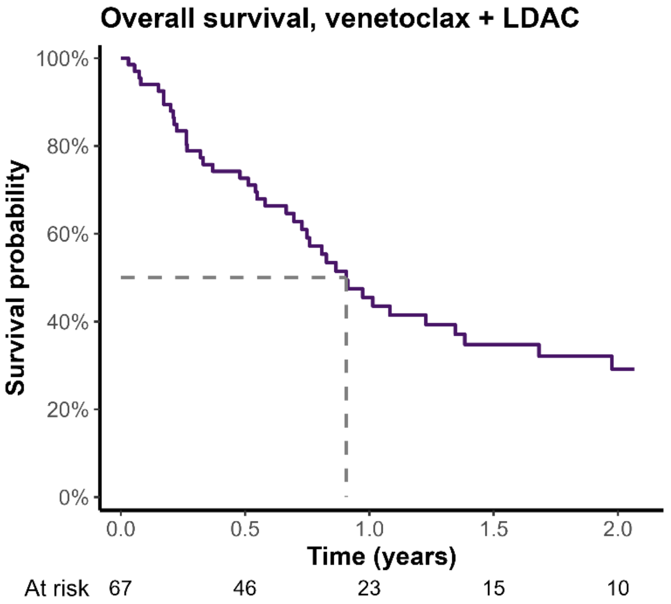

Figure S9 – overall survival in *NPM1* mutated AML

A. By presence of *FLT3* ITD

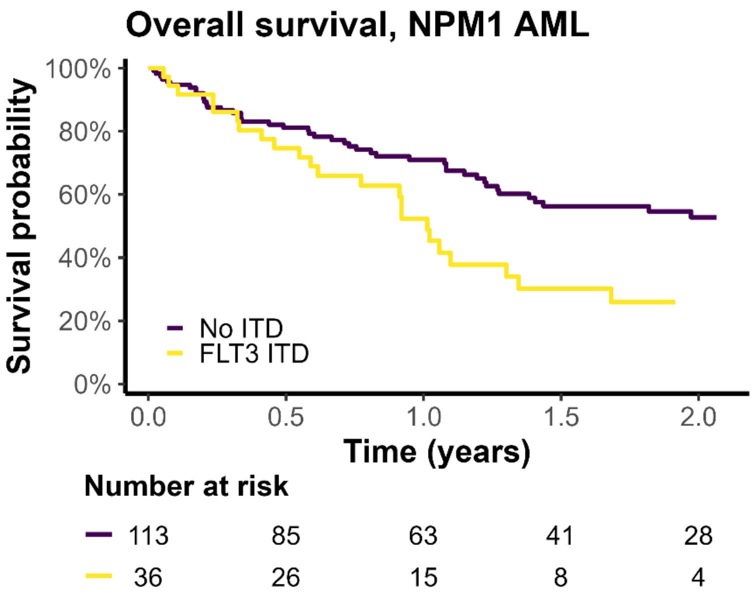

B. By *FLT3*-ITD and *DNMT3A* mutation

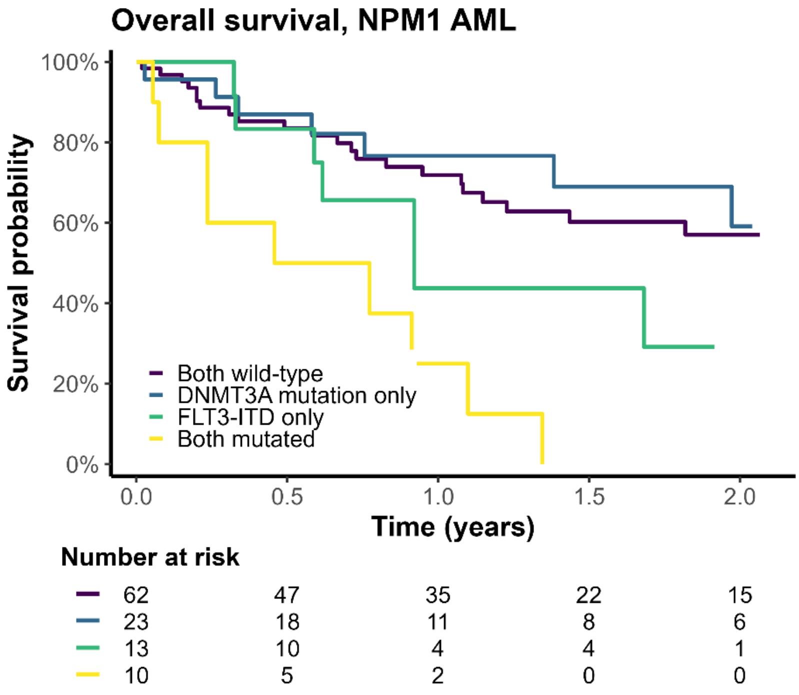

C. Comparing venetoclax and azacitidine with venetoclax and LDAC

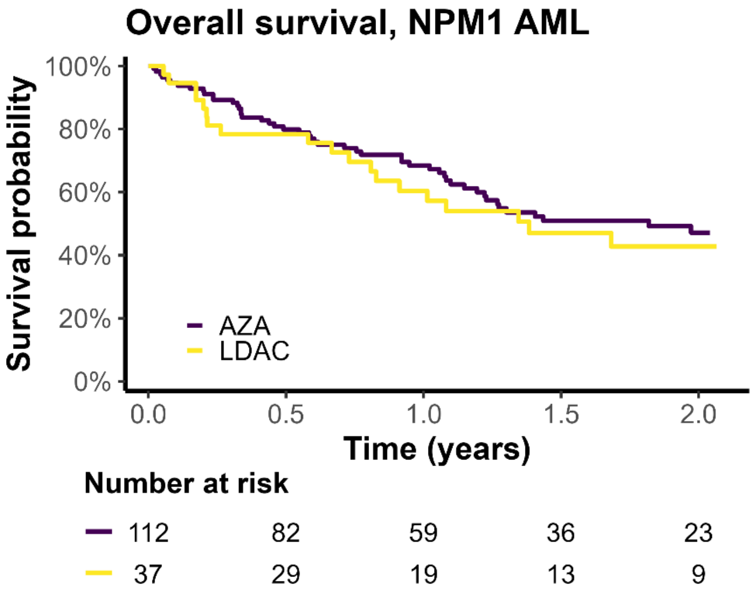

Supplement: Supplemental Methods, Tables, and Figures [file BNEO_NEO-2024-000274-mmc1.pdf]
